# A study on the apterous genus *Clytomelegena* Pic, 1928 (Coleoptera, Disteniidae)

**DOI:** 10.3897/zookeys.216.3769

**Published:** 2012-08-21

**Authors:** Meiying Lin, Sergey V. Murzin

**Affiliations:** 1 Key Laboratory of Zoological Systematics and Evolution, Institute of Zoology, Chinese Academy of Sciences, Beijing 100101; 2 8-1-23, Proletarsky prosp., Moscow, Russia

**Keywords:** *Clytomelegena* Pic, *Noeconia* Murzin, new synonym, new combination, new locality, Oriental region, Disteniidae

## Abstract

The genus *Noeconia* Murzin, 1988 is synonymized with *Clytomelegena* Pic, 1928. *Clytomelegena kabakovi* (Murzin, 1988), **comb. n.** is newly recorded from China (Guangxi Prov.). And Laos (Attapeu Prov.) is a new locality of this genus. Both sexes are apterous. Photographs and genitalic descriptions of *Clytomelegena kabakovi* are presented for the first time.

## Introduction

The first author, Meiying Lin found an old strange Distenidae specimen in the collection of the Chinese Agriculture University in Beijing in the past. Surprisingly in 2011, additional specimens collected at the same locality in China were brought to her by Messrs. Xinlei Huang and Huihua Zeng. It was first determined as *Noeconia kabakovi* Murzin, 1988 with the help of her European friends. Later, the second author, Sergey V. Murzin visited her Institute and informed her that *Noeconia kabakovi* could be a synonym of *Clytomelegena postaurata* Pic, 1928.

The genus *Clytomelegena* was first described by Pic (1928) on the basis of a unique specimen, *Clytomelegena postaurata* Pic, 1928 from South Vietnam. Later in 1988, Murzin described the genus *Noeconia* on the basis of *Noeconia kabakovi* Murzin, 1988 from North Vietnam.

According to our study of the type specimens of these two genera, it is concluded that the genus *Noeconia* should be a synonym of *Clytomelegena* by the resemblance of the structures of antennae, prothorax, elytra, abdomen and lack of hind wings. However, these two species are thought to be different species in spite of the problem that the type specimen of *Clytomelegena postaurata* seems to be a teneral individual.

As luck would have it, one specimen collected in Laos was brought to us as an addition to the abnormal unique type of *Clytomelegena postaurata*, which suggests that *Clytomelegena postaurata* and *Noeconia kabakovi* are surely different species, and the elytral shape could be a useful character to separate species. As a result, it is indicated that *Clytomelegena postaurata* is distributed in South Vietnam and Laos, and *Noeconia kabakovi* is distributed in North Vietnam and South China. The Chinese fauna of the Disteniidae is thus updated to 3 tribes, 8 genera and 28 species (Lin et al. 2010).

Types and other material studied are deposited in the following institutions or private collections:

CAU China Agricultural University, Beijing, China

CWD Private collection of Dong Wen, Qingdao, Shandong, China

EVC Eduard Vives collection, Terrass a, Spain

IEER A. N. Severtzov Institute of Ecology and Evolution (=IEMEA, A. N. Severtzov Institute of Evolutionary Morphology and Ecology of Animals), Moscow, Russia

IZAS Institute of Zoology, Chinese Academy of Sciences, Beijing, China

MNHN Muséum National d’Histoire Naturelle, Paris, France

NMPC National museum, Prague, Czech Republic

USNM National Museum of Natural History, Smithsonian Institution, Washington, DC, USA

ZIN Zoological Institute, Saint-Petersburg, Russia

## Results

### Genus Clytomelegena

Pic, 1928

http://species-id.net/wiki/Clytomelegena

Clytomelegena Pic, 1928: 11. Type-species: *Clytomelegena postaurata* Pic, 1928. Monotypy. —Villiers 1958: 267.
Noeconia Murzin, 1988: 161. syn. n. Type-species: *Noeconia kabakovi* Murzin, 1988. Original designation and monotypy.

#### Redescription.

Body small, slender; elytra not wider than prothorax at humeri and widened behind middle. Eyes finely faceted, oval; with very small emargination. Prothorax subequal to or more than 1.5 times as long as basal width, with round lateral tubercles behind middle; with a slight apical constriction, apical part subequal to or a little narrower than base. Scutellum pentagonal. Elytra depressed behind scutellum, swollen behind middle, evenly rounded apically. Hind wings reduced (Fig. 11; not mentioned in either of the original descriptions). Antennae thin, about 1.5 to 1.8 times as long as body, 3^rd^ to 10^th^ antennal joints internally with recumbent undulating long hairs, reaching the apex of corresponding joint. Procoxal cavity open behind (Fig. 14). Femora spindle-shaped, petiolate (Fig. 7); male hind femora reach elytral apex. Middle tibiae with an oblique groove bearing a brush of hairs. 1^st^ joint of posterior tarsi shorter than or subequal to following two joints together.

#### Remarks.

The genus belongs to the tribe Disteniini, close to *Nericonia* Pascoe and *Noemia* Pascoe, but differs by having the elytra swollen (Murzin 1988) and hind wings lacking. Our study of *Noeconia kabakovi* Murzin, 1988 revealed no huge high-level differences from *Clytomelegena postaurata* Pic, 1928, and therefore *Noeconia* Murzin, 1988 is herein synonymized with *Clytomelegena* Pic, 1928. It hosts two species up to now: *Clytomelegena postaurata* Pic, 1928 and *Clytomelegena kabakovi* (Murzin, 1988), comb. n.

Two flightless Oriental genera are known up to now: *Clytomelegena* Pic, 1928 and *Olemehlia* Holzschuh, 2011.

#### Distribution:

China (new country record): Guangxi Prov.; Laos (new country record): Attapeu Prov.; Vietnam: Vinh Phuc Prov., Batkhay Prov., Ninh Binh Prov. (Cuc Phuong National Park, new province record), Cao Bang Prov. (new province record), Lamdong Prov.

### Clytomelegena postaurata

Pic

http://species-id.net/wiki/Clytomelegena_postaurata

Figs 1–2

Clytomelegena postaurata Pic, 1928: 11.
Clytomelegena postaurata ; Villiers 1958: 267; Jiang and Wu 1986: 5.

#### Remarks.

**Figures 1–3.**
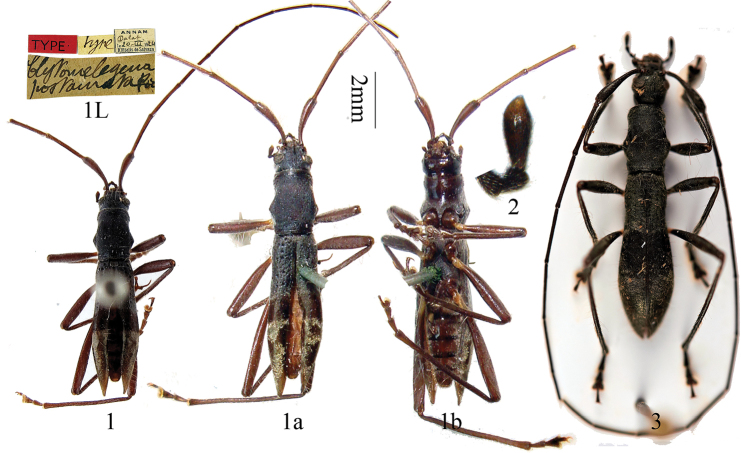
*Clytomelegena postaurata* Pic, 1928. **1** Holotype of *Clytomelegena postaurata* Pic, female, from Annam of Vietnam. **a** dorsal view **b** ventral view. L. labels **2** showing the shape of last segment of maxillary palp, in dorsal view **3** *Clytomelegena* sp., a female from Attapeu Prov. of Laos, similar to *Clytomelegena postaurata*. **1a** & **1b** scale 2 mm, others not to scale.

The unique type specimen deposited in MNHN is a newly emergent individual, which is difficult to compare with other specimens. The testaceous antennae and legs could be its color, or they would become as dark as the specimen from Laos or same to Murzin’s *kabakovi*. The elytra are not fully sclerotized, making specific character coding such as maculae unclear but revealing the absence of hind wings without opening the elytra. The last segment of maxillary palp (Fig. 2) and the last visible sternite (Fig. 1b) indicate that it is a female.

Measurement on the holotype: Elytra length: humeral width = ca. 3.5; pronotum length: pronotum maximum width = ca. 1.2; elytra length: prothorax length = ca. 2.7.

The relative length of antennal joints: 17:0.5:18:17:18:18:17:16:15:14:11.

#### Specimens examined.

Holotype (Fig. 1) of *Clytomelegena postaurata* Pic, female, Vietnam, Annam, Dalat (South Vietnam, LamDong Prov.) (MNHN, ex collection M. Pic).

#### Distribution:

Vietnam (LamDong Prov.).

### Clytomelegena sp.

Fig. 3

#### Remarks.

The female from Laos is more similar to Pic’s *postaurata* than Murzin’s *kabakovi* based on the measurement and the shape of elytral apex.

Measurement of the *Clytomelegena* sp. from Laos: Elytra length: humeral width = ca. 4.0; pronotum length: pronotum maximum width = ca. 1.3; elytra length: prothorax length = ca. 2.6.

#### Specimens examined.

1 female, Laos, Attapeu Prov., Annam Highlands Mts., Dong Amphan NBCA, ca 1,160 m, Nong Fa (Crater Lake env.), 15°05.9'N, 107°25.6'E, 2010.IV.30–V.6, Jiři Hájek leg (NMPC).

### Clytomelegena kabakovi

(Murzin) comb. n.

http://species-id.net/wiki/Clytomelegena_kabakovi

Figs 4 [Fig F3] –27

Noeconia kabakovi Murzin, 1988: 162, fig. 1. — Jeniš 2001: 17, Pl. III, fig. 22.

#### Redescription of species.

Body length 8.8–14.5 mm, width at humeri 1.7–2.2 mm. Black; trochanters and bases of femora yellow; mouth parts, most parts of legs and antennae brownish. Body with long scattered erect setae and recumbent silvery pubescence, which is sparser on head and abdominal sternites and absent on ventral sides of head and prothorax. Elytra with a transverse band behind middle created by recumbent dark-brown setae and pubescence, of which width is about 1/4 of elytral length.

Head finely irregularly rugose between eyes, with longitudinal rugose sculpture under eyes and behind antennal bases. The last segment of maxillary palp stout (male, Fig. 8) or slender (female, Fig. 9). Pronotum longer than broad, with very fine and dense punctation. Elytra with 4 longitudinal rows of punctures, which are deep and hollow-shaped anteriorly and missing at middle, with very fine indistinct sculpturing between punctures, independently rounded apically, with a row of 7–10 pointed tubercles behind humeri. Antennae long and thin, 1.5 (female) to 1.8 (male) times longer than body; Scape dilated toward apex, reaching midlength of pronotum. Pedicel very small, spherical, hidden inside apical hollow of scape. The relative length of antennal joints (male and female almost same): 20:1:19:17:18:18:17:16:15:13:14.

**Figures 4–14.**
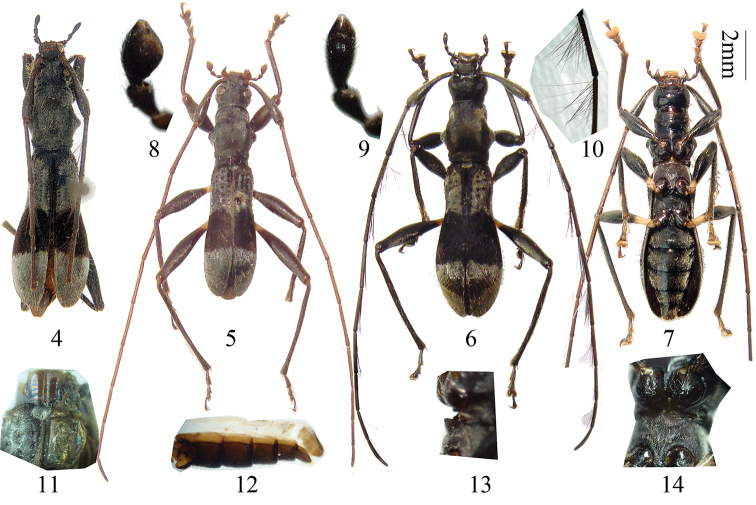
*Clytomelegena kabakovi* (Murzin, 1988). **4–7** Habitus. **4** female, from Tonkin of Vietnam **5** male, from Guangxi of China **6** female in dorsal view, from Guangxi of China **7** female in ventral view, from Guangxi of China. **8–9** showing last segment of maxillary palp. **8** male **9** female. **10** showing the fringed long hairs of antennomeres **11** showing mesonotum with a median groove and hind wings reduced (right elytron removed) **12** lateral view of male abdomen, showing membranous tergites (except tergite VII) **13** mesosernum in lateral view, showing median basal part protruding **14** procoxal cavity, open posteriorly. **4–7** Scale 2 mm. **8–14** not to scale.

Measurement on male: Elytra length: humeral width = ca. 3.1; pronotum length: pronotum maximum width = ca. 1.1; elytra length: prothorax length = ca. 2.5.

Measurement on the female: Elytra length: humeral width = ca. 3.2; pronotum length: pronotum maximum width = ca. 1.1; elytra length: prothorax length = ca. 2.6.

#### Male terminalia

**Figures 15–23. F3:**
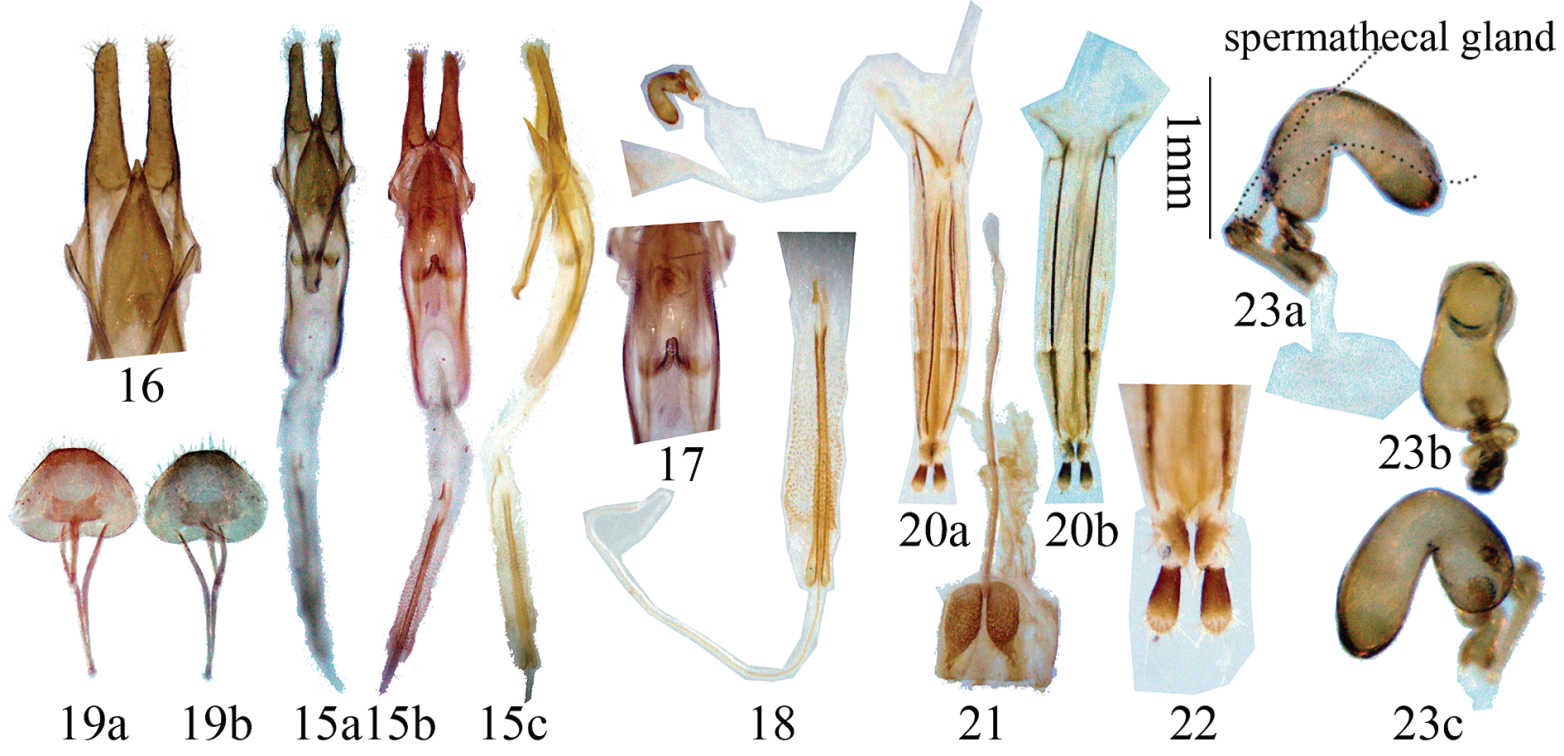
Genitalia of *Clytomelegena kabakovi* (Murzin, 1988). **15–19** male. **15** male genitalia **16** showing lateral lobes and apex of ventral plate **17** showing basal armature in intersal sac **18** showing rods of endophallus and ejaculatory duct **19** tergite VIII and sternites VIII & IX in ventral view. **a** ventral view **b** dorsal view **c** lateral view. **20–23** female. **20** female genitalia. **a** dorsal view **b** ventral view **21** tignum, ventral view **22** showing stylus **23** spermathecal capsule in different views. **15, 19, 20, 21** scale 1 mm, others not to scale.

**(Figs 15–19):** Tegmen approximately 1.6 mm in length; lateral lobes slender, approximately 0.5 mm long and 0.1 mm wide, apex with short setae; median lobe plus median struts slightly curved, subequal to tegmen in length; the median struts less than 1/3 of the whole median lobe in length; dorsal plate shorter than ventral plate; apex of ventral plate sharply pointed; internal sac moderately long, about twice the median lobe in length, bearing a basal armature (Fig. 17) and two apical rods of endophallus (Fig. 18), ejaculatory duct single (Fig. 18). Apex of tergite VIII truncated with rounded sides (Fig. 19). Female terminalia (Figs 20–23): Paraproct moderate in size, its baculi thick and long, straight and not bifurcate at base; valvifer baculum very thick at base and narrowed towards apex (Fig. 20b); coxite lobes sclerotized at each inner part, with tactile hairs; stylus articulated to the tip of each coxite lobe, sclerotized except for apex and bearing tactile hairs (Fig. 22); dorsal baculi straight and longer than paraproct baculi (Fig. 20a); proctiger baculi long and almost straight (Fig. 20a). Spermathecal capsule (Fig. 23) is complex and coiled, composed of two parts, with two openings to bursa copulatrix (or spermathecal duct); bigger one with basal 1/4 twisted, strongly curved near middle; the other small part also strongly sclerotized, curved and twisted at middle, connected with bigger part with a thin duct; spermathecal gland (Fig. 23a) attached to middle of smaller part of capsule, membranous. Tignum (Fig. 21) slightly longer than half of abdomen. In one measured specimen, tignum was 2.3 mm for an adult with 4.2 mm abdomen length in ventral view.

#### Biology and ecology:

**Figures 24–27.**
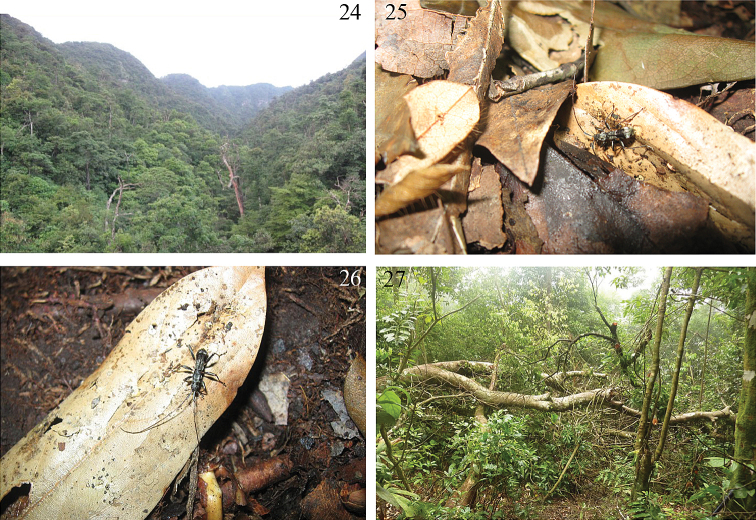
Biotope of *Clytomelegena kabakovi* (Murzin, 1988). **24** tropical rainforest of collecting site in Guangxi, China (by Huihua Zeng) **25–26** crawling on the leaf litter near light trap site (by Huihua Zeng) **27** collecting site in Tam Dao National Park of North Vietnam (by E. Vives).

Prior to this study, no biological or ecological information was published on this species. The second author S. Murzin collected some specimens of this species in Cuc Phuong National Park (N. Vietnam) on 3–5 May 1991 on leaves of different plants. On 5 June 2011, Huihua Zeng collected one specimen on the ground near a light trap, but it was not certain whether this was an accidental occurrence or whether the specimen was attracted by light. Later (9 July 2011), the same collector observed another specimen on a stump near the light trap, later crawling in the leaf litter on the ground (Figs 24–26). The light trap was located in Damingshan of Guangxi, a tropical rainforest, at the altitude of 1,200 m. The other collector, Xinlei Huang, also collected one specimen at the same locality, by sweep net, which likewise did not elucidate any information on its biology.

Eduard Vives collected one female on 14 June 2011 in Tam Dao National Park of North Vietnam. It was crawling in a very antlike manner on the trunk of a large, recently fallen tree (Fig. 27) that was also attracting many *Agrilus* (Buprestidae) and small Lamiinae (genus *Sybra*, *Pterolophia*, *Exocentrus*). The day was very sunny and Eduard watched this trunk for 80 minutes more and did not see any additional specimens of *Clytomelegena* (personal communication, Aug. 2011).

In 2012, 3 additional specimens were collected in North Vietnam from Cao Bang Province and Ninh Binh Province in April and May by an expedition of Steven Lingafelter, Eduard Jendek, and Pham Hong Thai.

#### Remarks.

We consider Murzin’s *kabakovi* a different species from Pic’s *postaurata* based on the following reasons:

1) Elytron with a row of 7–10 pointed tubercles behind humeri, while tubercles missing in *Clytomelegena postaurata* (based on the unique type specimen);

2) Elytral apex broader (bluntly rounded instead of sharply rounded) and elytra shorter (the ratio of elytral length to basal width smaller);

3) Antennomere XI slight longer than antennomere X (much shorter in *Clytomelegena postaurata*)

4) Hairs on elytra much shorter;

5) Pronotum with bigger lateral tubercles and swellings on the sides of the disc not as flattened as that on *Clytomelegena postaurata*;

6) Width of the dark-brown transverse band behind middle of elytra is about 1/4 of elytral length, while in *Clytomelegena postaurata* is only 1/6.

This species is recorded from China for the first time. It is the 28^th^ recorded species for the Chinese Disteniidae fauna.

#### Distribution:

China (new country record): Guangxi Prov.; Vietnam: Vinh Phuc Prov., Batkhay Prov., Ninh Binh Prov. (Cuc Phuong National Park, new province record), Cao Bang Prov. (new province record).

#### Specimens examined.

Type series of *Noeconia kabakovi* Murzin, 1988. Holotype, male, Batkhay Prov.: mountains in 50 km N-E Tkhaynguen, 1963.V.14, coll. O.N. Kabakov; paratype, 1 female, (Bakthai Prov.), distr. Fulyong, village Kuangtchu, 1986.IV.23, coll. A.V. Sharkov; paratype, 1 female, Prov. Vin’fu; Tamdao, alt. 800m, 1962.V.14, coll. O.N.Kabakov. All the types were preserved in the collections of IEER and ZIN.

**Specimens from China, Guangxi:** 1 male, Nanning, Wuming county, Mt. Damingshan, 1963.V.21, coll. Jikun Yang (CAU); 1 female, Nanning, Wuming county, Mt. Damingshan, Tianping station, alt. 900–1,260 m, 23.51770°N, 108.39295°E, 2011.V.23, by sweeping net, coll. Xinlei Huang (IZAS); 1 female, Nanning, Wuming county, Mt. Damingshan, alt. 1200 m, 2011.VI.5, coll. Huihua Zeng (CWD); 1 female, same data but 2011.VII.9 (CWD).

**Specimen from North Vietnam:** 1 female, Vietnam North, Prov. Vinh Phuc, Tam Dao National Park, 1,100 m, 2011.VI.14, coll. Eduard Vives (EVC); 2 females, Cao Bang Prov., Phja-Den Environs, 22°32.433'N, 105°52.012'E, alt. 948 m, 2012.V.2, day collecting, coll. Steven Lingafelter, Eduard Jendek, Pham Hong Thai (USNM); 1 female, Ninh Binh Prov., Cuc Phuong National Park, 20°21.012'N, 105°35.592'E, alt. 439 m, 2012.IV.25, day collecting, coll. Steven Lingafelter, Eduard Jendek, Pham Hong Thai (USNM).

## Supplementary Material

XML Treatment for Clytomelegena

XML Treatment for Clytomelegena postaurata

XML Treatment for Clytomelegena

XML Treatment for Clytomelegena kabakovi
